# Supplementary material for: Causal Inference and Shared Molecular Pathways in Crohn’s Disease, Celiac Disease, and Ankylosing Spondylitis: Integrative Mendelian Randomization and Transcriptomic Analysis
Source: Int J Mol Sci. 2025 Jul 4;26(13):6451. doi: 10.3390/ijms26136451 (PMC12249856; doi:10.3390/ijms26136451)

**Supplementary File S10.** Gene-miRNA interactions	
Hub genes	miRNA
P2RY8	hsa-miR-34a-5p; hsa-miR-152-3p; hsa-mir-373-3p; hsa-mir-1271-3p; hsa-miR-107; hsa-miR-148a-3p; hsa-miR-27a-3p; hsa-mir-146a-5p; hsa-miR-27b-3p; hsa-miR-103a-3p; hsa-mir-191-5p; hsa-mir-142-5p; hsa-miR-15a-5p
ITGAL	hsa-miR-34a-5p; hsa-mir-191-5p; hsa-miR-196b-5p; hsa-let-7b-5p; hsa-mir-93-3p; hsa-miR-3074-5p; hsa-miR-576-3p; hsa-let-7d-5p; hsa-miR-181c-5p; hsa-miR-365a-5p; hsa-miR-181b-5p; hsa-mir-4651; hsa-mir-342-5p; hsa-miR-4745-5p; hsa-miR-3940-5p; hsa-miR-196a-5p; hsa-miR-26a-5p; hsa-miR-26b-5p
GPR65	hsa-miR-34a-5p; hsa-miR-26b-5p; hsa-miR-26a-5p; hsa-miR-196a-5p; hsa-mir-142-5p; hsa-miR-15a-5p; hsa-miR-130b-3p; hsa-miR-106b-5p; hsa-miR-20b-5p; hsa-miR-450a-5p; hsa-miR-301a-3p; hsa-miR-625-5p; hsa-mir-429; hsa-miR-33b-5p; hsa-miR-296-3p; hsa-let-7a-5p; hsa-miR-19a-3p; hsa-miR-155-5p; hsa-miR-17-5p hsa-miR-19b-3p; hsa-miR-148a-5p; hsa-let-7i-5p; hsa-let-7a-3p; hsa-miR-30c-2-3p; hsa-mir-20a-5p; hsa-miR-138-2-3p; hsa-let-7g-5p; hsa-miR-106a-5p; hsa-mir-203a-3p; hsa-mir-9-3p; hsa-mir-1270; hsa-mir-1343-3p; hsa-miR-33a-5p

**Supplementary File S10.** Transcription factor–gene interactions	
Hub genes	Transcription factor
P2RY8	WRNIP1; MLLT1; YBX1; EBF1; DMAP1; EED; BCL11A; TBX21; ZNF366; CBFB; NFIC; POU2F2; HDGF
ITGAL	WRNIP1; SUPT5H; YBX1; EBF1; DMAP1; EED; KLF7; MXD4; SSRP1; KDM1A; THRB; KLF9; ZBTB26; RARA; ZNF18; CEBPA; ZNF644; BCOR; MLX; ATF1; BCL6; ZHX2; NFIA; KLF11; MYB; RXRB
GPR65	WRNIP1; MLLT1; SUPT5H; ZNF83; SMARCA4; GATA2; SIRT6

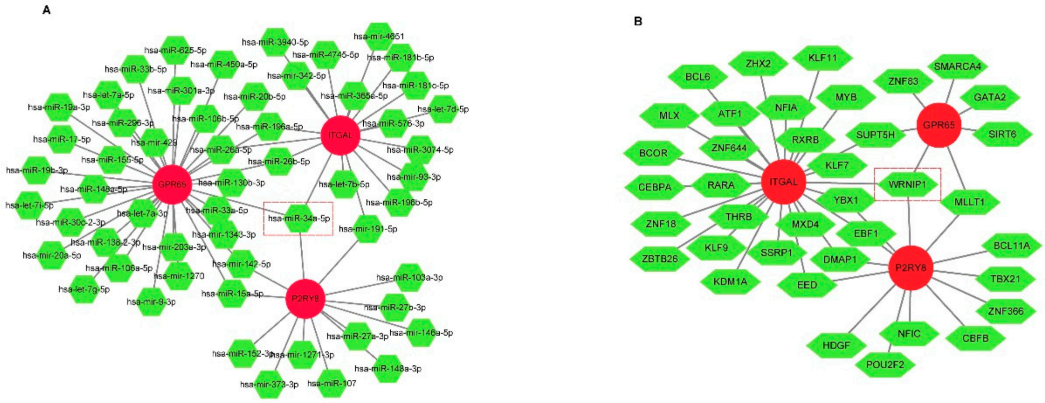

Supplement: Supplementary file 1 [file ijms-26-06451-s001.zip › Supplementary File S10.pdf]
