# Supplementary material for: Causal Inference and Shared Molecular Pathways in Crohn’s Disease, Celiac Disease, and Ankylosing Spondylitis: Integrative Mendelian Randomization and Transcriptomic Analysis
Source: Int J Mol Sci. 2025 Jul 4;26(13):6451. doi: 10.3390/ijms26136451 (PMC12249856; doi:10.3390/ijms26136451)

Supplementary File S11. SDS-PAGE（The black-boxed section in the figure is shown in the main text as Figure 7C.）

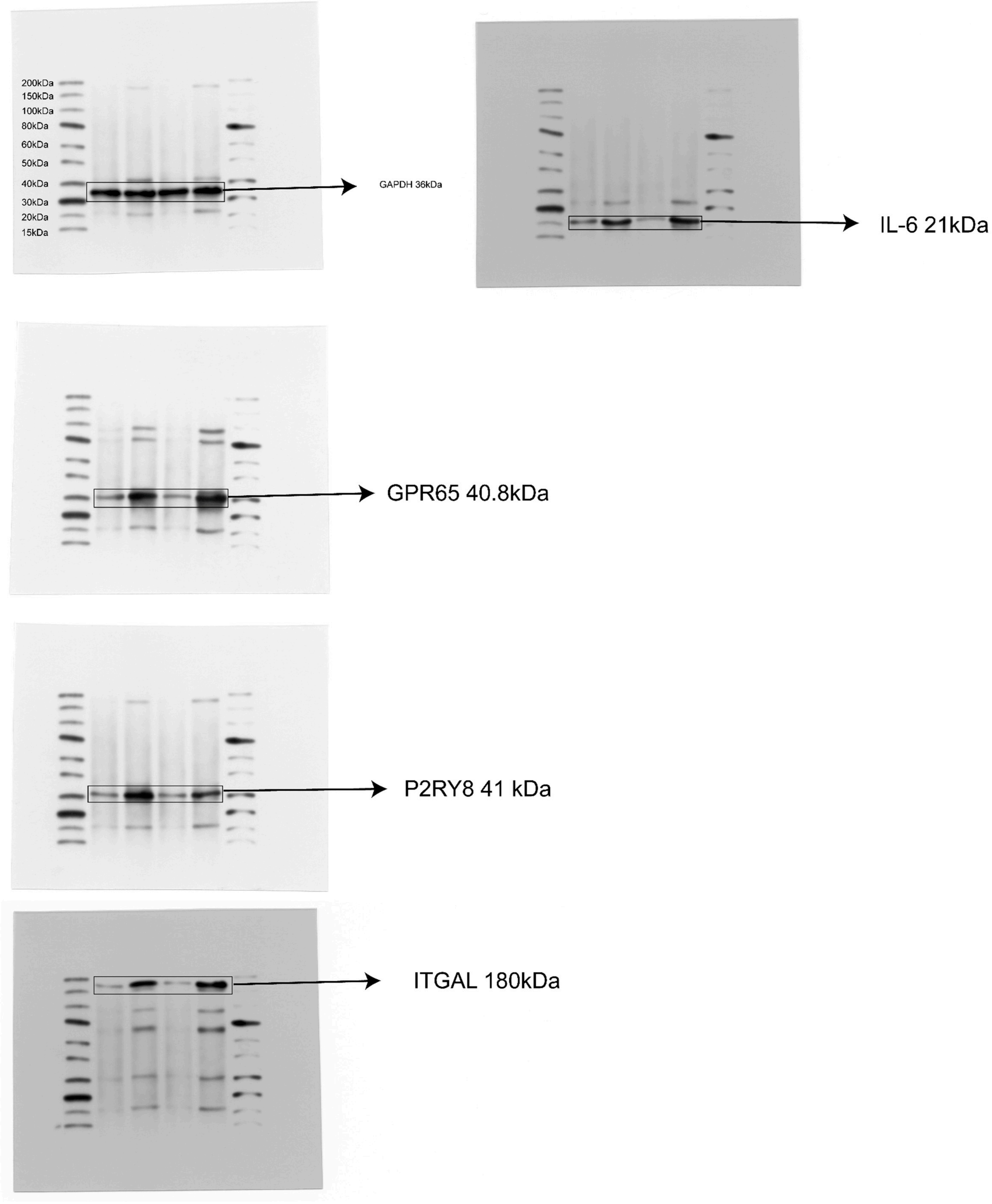

To ensure the accuracy of the Western blot (WB) results and to prevent signal interference due to the close molecular weights of P2RY8 (40.8 kDa) and GPR65 (41 kDa), we repeated the WB experiment for GPR65 to independently verify its protein expression level.

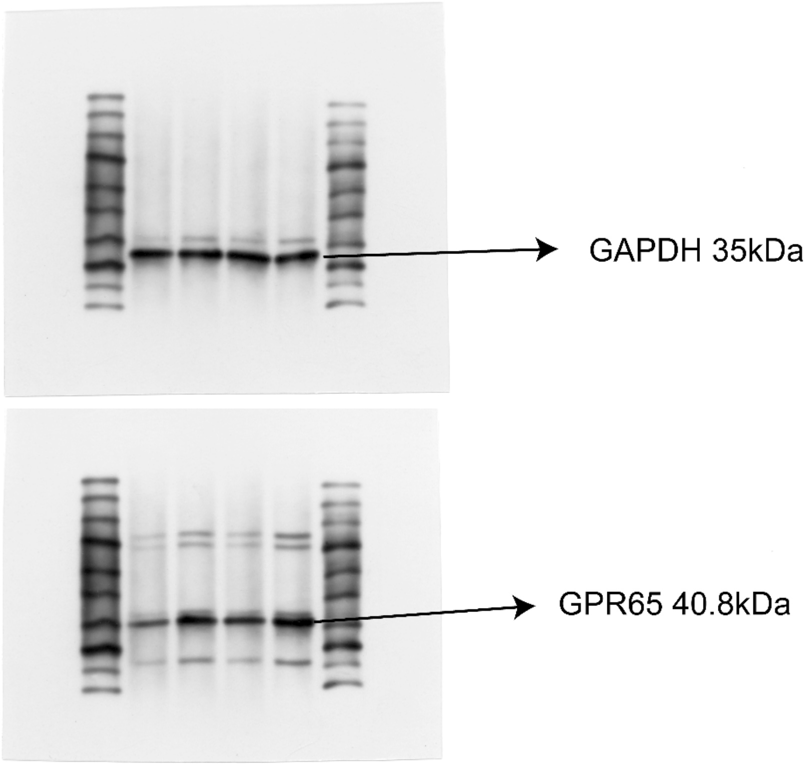

Supplement: Supplementary file 1 [file ijms-26-06451-s001.zip › Supplementary File S11.pdf]
