# Supplementary material for: Causal Inference and Shared Molecular Pathways in Crohn’s Disease, Celiac Disease, and Ankylosing Spondylitis: Integrative Mendelian Randomization and Transcriptomic Analysis
Source: Int J Mol Sci. 2025 Jul 4;26(13):6451. doi: 10.3390/ijms26136451 (PMC12249856; doi:10.3390/ijms26136451)
Supplement: Supplementary file 1 [file ijms-26-06451-s001.zip › Supplementary File S3.pdf]

Supplementary File S3. Sensitivity Analyses and FDR Correction for Mendelian Randomization Results

Exposure	Outcome	MR-Egger intercept analysis		Heterogeneity tests by IVW		Heterogeneity tests by MR-Egger	
		Intercept	Intercept p-value	Cochran's Q	p-value	Cochran's Q	p-value
Crohn disease	Ankylosing spondylitis	0.007	0.872	2.845	0.416	11.746	0.228
Ankylosing spondylitis	Crohn disease	0.018	0.459	21.871	0.528	16.369	0.839
Crohn disease	Celiac disease	0.012	0.901	2.974	0.395	2.945	0.229
Celiac disease	Crohn disease	0.051	0.241	10.929	0.449	9.380	0.496
Ankylosing spondylitis	Celiac disease	0.0003	0.991	23.131	0.337	14.417	0.851
Celiac disease	Ankylosing spondylitis	0.0072	0.603	6.461	0.693	14.854	0.095

In heterogeneity analysis, a p-value < 0.05 indicates significant heterogeneity, whereas in pleiotropy analysis, it suggests the presence of significant horizontal pleiotropy.

Exposure	Outcome	IVW p-value	IVW FDR	MR Egger p-value	MR Egger FDR	Weighted median p-value	Weighted median FDR	Simple mode p-value	Simple mode FDR	Weighted mode p-value	Weighted mode FDR
Crohn's disease	Ankylosing spondylitis	7.80E-06	4.68E-05	0.753281976	0.812	1.23E-06	7.38E-06	0.03626624	0.2048	0.02686408	0.0806
Ankylosing spondylitis	Crohn's disease	0.005447908	0.01634	0.338264218	0.8057	0.153445149	0.1841	0.186786906	0.2802	0.022350403	0.0806
Crohn's disease	Celiac disease	0.015704568	0.03141	0.812000854	0.812	0.288918825	0.2889	0.423752694	0.4238	0.413462936	0.4135
Celiac disease	Crohn's disease	0.032112772	0.03854	0.537109955	0.8057	0.098390894	0.1476	0.377247987	0.4238	0.347638449	0.4135
Ankylosing spondylitis	Celiac disease	0.021458159	0.03219	0.441645034	0.8057	0.080107246	0.1476	0.119350626	0.2387	0.090769435	0.1362
Celiac disease	Ankylosing spondylitis	0.039087676	0.03909	0.115753461	0.6945	0.008280114	0.0248	0.068279734	0.2048	0.049830536	0.0997

SNP	EA	OA	id.exposure	RSSobs	p-value
rs12119179	C	A	finn-b-K11_CROHN	1.312E-06	1
rs55904328	A	G	finn-b-K11_CROHN	1.123E-06	1
rs6584283	C	T	finn-b-K11_CROHN	9.025E-06	1
rs73099728	C	T	finn-b-K11_CROHN	1.099E-05	1
rs1041926	A	G	ebi-a-GCST005529	0.0001447	1
rs11065898	T	C	ebi-a-GCST005529	2.177E-05	1
rs11190133	T	C	ebi-a-GCST005529	2.122E-05	1
rs11209026	A	G	ebi-a-GCST005529	0.0001355	1
rs1128905	C	T	ebi-a-GCST005529	3.443E-06	1
rs11624293	C	T	ebi-a-GCST005529	0.0029873	0.6
rs1250550	A	C	ebi-a-GCST005529	3.217E-05	1
rs12615545	C	T	ebi-a-GCST005529	2.758E-05	1
rs1801274	G	A	ebi-a-GCST005529	5.555E-06	1
rs1860545	A	G	ebi-a-GCST005529	4.397E-05	1
rs2517655	T	C	ebi-a-GCST005529	3.581E-06	1
rs2531875	T	G	ebi-a-GCST005529	6.421E-05	1
rs2596501	T	C	ebi-a-GCST005529	0.0008992	1
rs27529	G	A	ebi-a-GCST005529	4.966E-06	1
rs2836883	A	G	ebi-a-GCST005529	1.188E-05	1
rs35164067	A	G	ebi-a-GCST005529	0.0011218	0.375
rs4129267	T	C	ebi-a-GCST005529	0.0002576	1
rs41299637	G	T	ebi-a-GCST005529	0.0006979	1
rs4672505	G	A	ebi-a-GCST005529	0.0065474	0.975
rs4676410	A	G	ebi-a-GCST005529	0.0002266	1
rs6556416	C	A	ebi-a-GCST005529	0.0002302	1
rs6600247	C	T	ebi-a-GCST005529	0.0004338	1
rs7191548	C	T	ebi-a-GCST005529	1.135E-05	1
rs9901869	A	G	ebi-a-GCST005529	1.123E-05	1
rs1018326	C	T	ebi-a-GCST000612	9.371E-06	1
rs11221335	C	T	ebi-a-GCST000612	9.716E-05	1
rs13003464	G	A	ebi-a-GCST000612	3.466E-05	1
rs13098911	T	C	ebi-a-GCST000612	0.0002766	1
rs13151961	G	A	ebi-a-GCST000612	3.44E-05	1
rs1464510	A	C	ebi-a-GCST000612	4.345E-06	1
rs1738074	C	T	ebi-a-GCST000612	0.0002585	1
rs17810546	G	A	ebi-a-GCST000612	9.547E-05	1
rs2327832	G	A	ebi-a-GCST000612	8.932E-05	1
rs2816316	A	C	ebi-a-GCST000612	2.125E-05	1
rs653178	T	C	ebi-a-GCST000612	3.978E-05	1
rs917997	C	T	ebi-a-GCST000612	0.0005249	1

In the Outlier Test (MR-PRESSO), RSSobs refers to the observed value of the Residual Sum of Squares (RSS) for an SNP relative to the main regression line.It measures the deviation of the SNP effect from the overall model fit, with a p-value < 0.05 indicating the presence of horizontal pleiotropy.
