# Supplementary material for: Causal Inference and Shared Molecular Pathways in Crohn’s Disease, Celiac Disease, and Ankylosing Spondylitis: Integrative Mendelian Randomization and Transcriptomic Analysis
Source: Int J Mol Sci. 2025 Jul 4;26(13):6451. doi: 10.3390/ijms26136451 (PMC12249856; doi:10.3390/ijms26136451)
Supplement: Supplementary file 1 [file ijms-26-06451-s001.zip › Supplementary File S4.pdf]

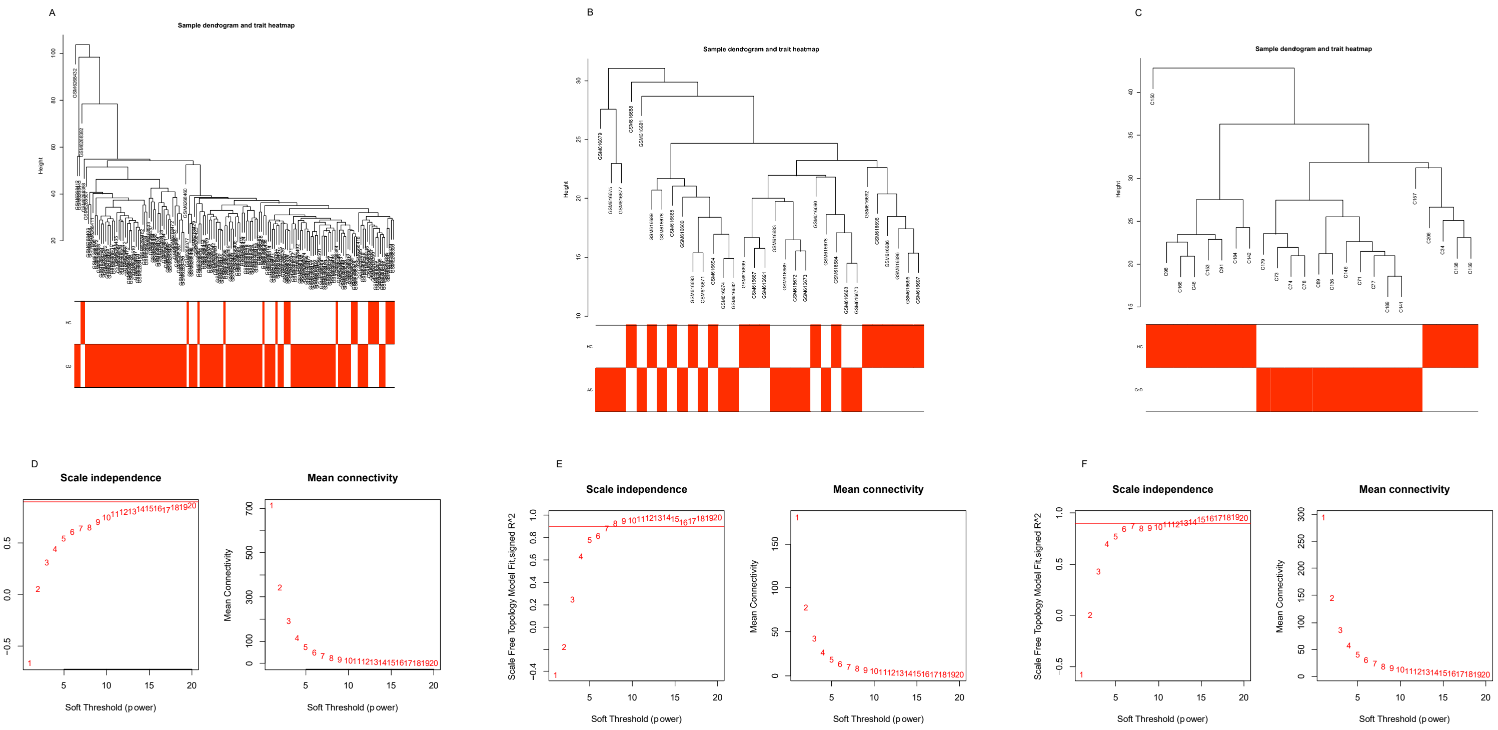

Supplementary File S4. Clustering analysis was performed to identify outliers, with all samples falling within the clusters and meeting the cutoff criteria. **(A)**GSE207022(CD) **(B)**GSE25101(AS) **(C)**GSE112102(CeD). Scale-free topology model fit and mean connectivity analysis for the WGCNA network construction in CD **(D)**, AS **(E)**, and CeD **(F)**. The x-axis represents the soft-thresholding power (β), and the y-axis shows the scale-free topology fit index (left) and mean connectivity (right). The optimal soft-thresholding power was selected as the lowest value at which the scale-free topology fit index exceeds 0.85 while maintaining a reasonable mean connectivity. CD: Crohn's disease; AS: Ankylosing spondylitis; CeD: Celiac disease
