# Supplementary material for: Causal Inference and Shared Molecular Pathways in Crohn’s Disease, Celiac Disease, and Ankylosing Spondylitis: Integrative Mendelian Randomization and Transcriptomic Analysis
Source: Int J Mol Sci. 2025 Jul 4;26(13):6451. doi: 10.3390/ijms26136451 (PMC12249856; doi:10.3390/ijms26136451)
Supplement: Supplementary file 1 [file ijms-26-06451-s001.zip › Supplementary File S5.pdf]

A

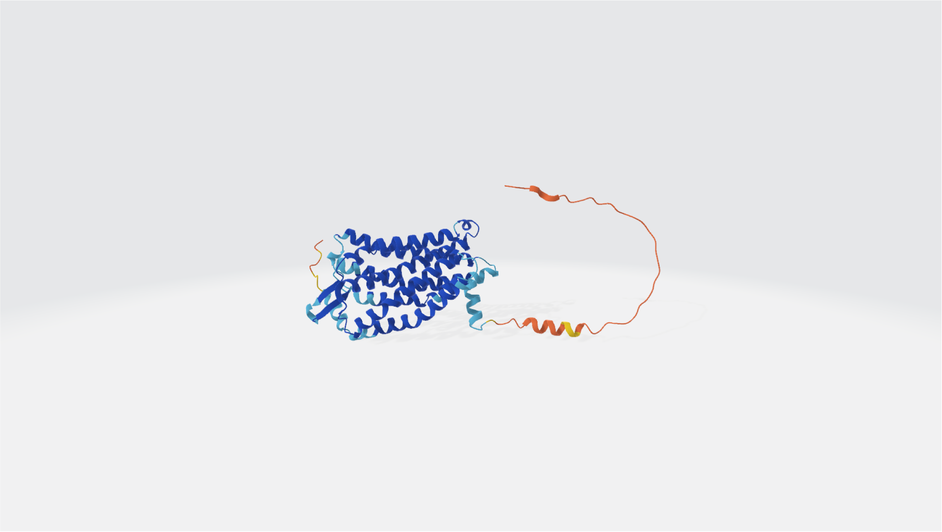

B

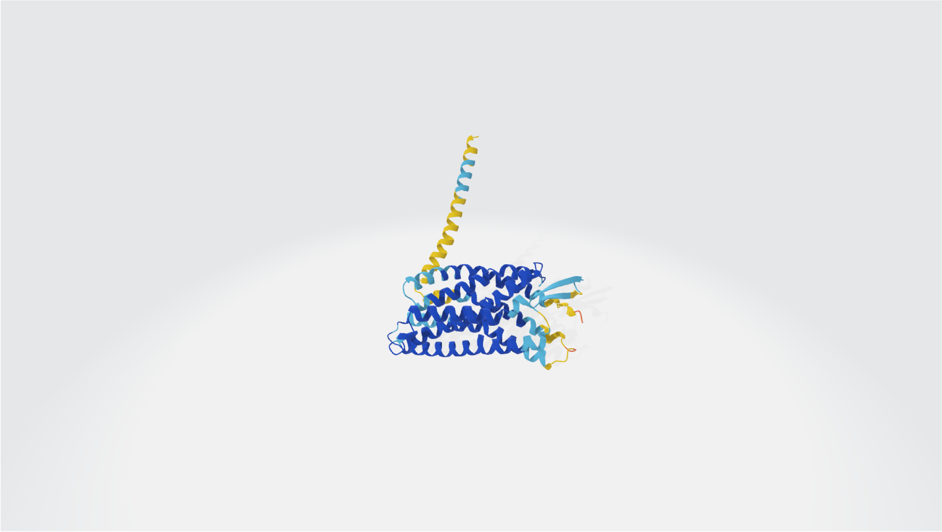

C

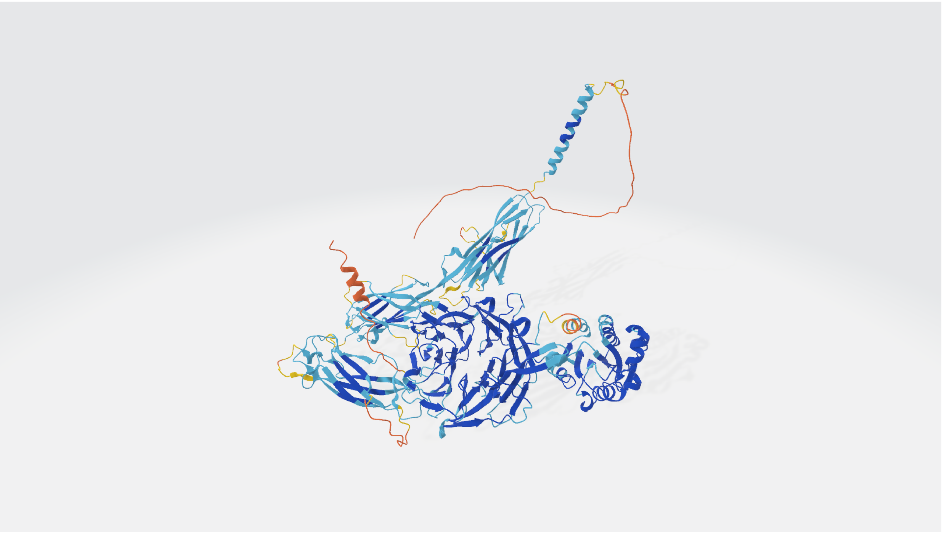

Supplementary File S5. Predicted Protein Structures.
Predicted 3D structures of P2RY8 (A), ITGAL (B), and GPR65 (C) using AlphaFold.
